# An inventory of ready-to-use and publicly available tools for the safety assessment of nanomaterials

**DOI:** 10.1016/j.impact.2018.08.007

**Published:** 2018-10

**Authors:** A. Paula K. Jantunen, Stefania Gottardo, Kirsten Rasmussen, Hugues P. Crutzen

**Affiliations:** European Commission, Joint Research Centre, Via E. Fermi 2479, I-21027 Ispra, Italy

**Keywords:** Nanomaterial, Safety assessment, Tools, Inventory, Regulation

## Abstract

Legislation addressing environmental, health and safety aspects of nanomaterials in consumer products and ensuring their safe use is being continuously updated in the European Union and globally. This leads to a growing need for tools to implement this developing legislation. A freely accessible inventory of ready-to-use and publicly available tools that together cover the tasks within a nanomaterial safety assessment process was built in the presented work. This inventory is a unique metadata set in Excel® format: the ‘NANoREG Toolbox’, which assembles information needed for selecting and accessing instruments that meet specific goals. The recorded tools are categorised according to their purpose, type and regulatory status. The Toolbox covers an unprecedented and broad range of over 500 current tools, developed in Europe and beyond. While NANoREG focussed on safety assessment under the EU Regulation on Registration, Evaluation, Authorisation and Restriction of Chemicals (REACH), the instruments in the Toolbox are relevant and useful for nanomaterial safety assessments worldwide.

## Highlights

• We present a large inventory of published and ready-to-use safety assessment tools
• The 544 tools included cover all tasks within a nanomaterial safety assessment
• The unique ‘NANoREG Toolbox’ metadata set is freely accessible
• The Toolbox is relevant and useful for nanomaterial assessments globally

## Graphical abstract

**Figure:**
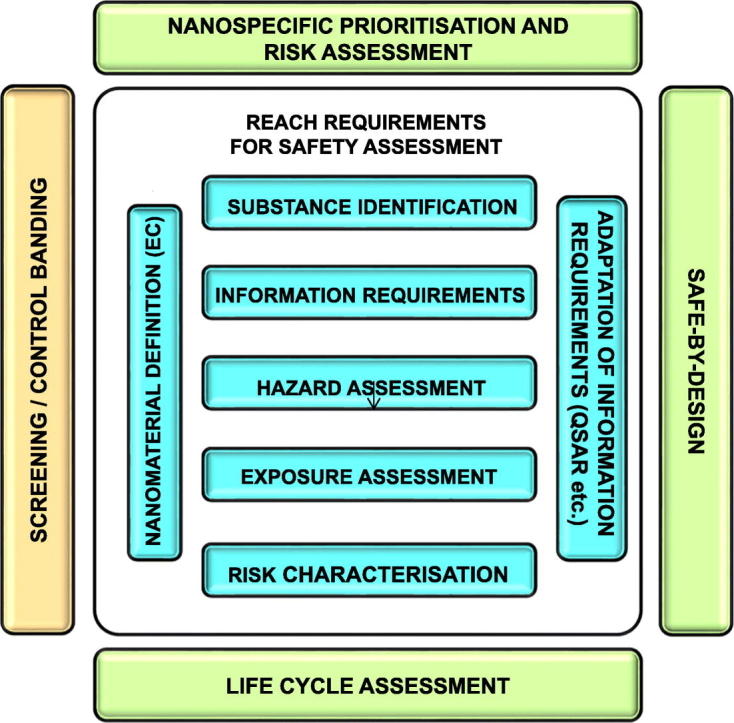

## 1 Introduction

Environmental, Health and Safety (EHS) aspects of nanotechnology applications and nanomaterials (NMs) have been debated in the scientific and regulatory communities since the early 2000s (e.g. Oberdörster et al., 2005; NSTC, 2006; Maynard et al., 2006; Science Policy Group Council, 2007; EC, 2008; Warheit, 2008; Warheit et al., 2008). Several scientific reviews (e.g. Grieger et al., 2012; Hristozov et al., 2012; Gottschalk et al., 2013; Praetorius et al., 2014; Hartmann et al., 2015; McClements & Xiao, 2017; Guinée et al., 2017) and roadmaps for research and legislation (e.g. EC, 2004; National Science and Technology Council (NSTC), 2008, National Science and Technology Council (NSTC), 2011; NRC, 2012; Savolainen et al., 2013; Stone et al., 2014, Stone et al., 2017) have summarised knowledge, listed tools, identified gaps, established priorities and made efforts to ensure that the research field of nanomaterial EHS (nanoEHS) evolves as quickly and robustly as possible, while informing and supporting the policy-making process and the adaptation of existing legislation to NMs. Recently, scientists have called for more adaptive, integrative and comparative risk governance of nanotechnologies, where anticipatory activities by regulators (e.g. horizon scanning), stakeholder involvement and public engagement are improved. Furthermore, it has been proposed that regulatory risk assessment be combined with flexible, predictive and semi-quantitative strategies, such as safe-by-design, grouping, control banding and decision analysis. This combination would enable early-stage assessment and management of NMs in a context of limited information and uncertainty (e.g. IRGC, 2007; Gottardo et al., 2017; ProSafe, 2017; Linkov et al., 2018; Stone et al., 2018).

In Europe, the European Commission has contributed to the generation of data, information and tools for enabling nanoEHS assessment and management by funding a number of research projects annually since 2005. The resulting increase of knowledge and awareness of scientific and regulatory hurdles has led to the introduction of legal definitions of the term ‘nanomaterial’ and the amendment of consumer protection legislation (concerning the use of NMs in e.g. cosmetics, food, food contact materials, biocides and medical devices) (Rauscher et al., 2017).

The European Union (EU) has indeed been the first region globally to lay down legally binding NM definitions, inspired by the horizontal definition recommended by the European Commission in 2011 (‘EC Recommendation’) (EC, 2011). The EC Recommendation is presently being reviewed by the European Commission in a stepwise process started by the Joint Research Centre (JRC) (Rauscher et al., 2014; Roebben et al., 2014; Rauscher et al., 2015), which may result in a more precisely worded definition. In addition, the EU Member States Competent Authorities have agreed on amendments to the Annexes of the EU Regulation on Registration, Evaluation, Authorisation and Restriction of Chemicals (REACH) (European Parliament and Council, 2006). These changes are to include a legally binding definition, based on the EC Recommendation, and specific information requirements for NMs. The European Parliament and Council are currently scrutinising these amendments before adoption (EC, 2018a). Meanwhile, the European Chemicals Agency (ECHA) has developed guidance for industry on how to register NMs (ECHA, 2017a) and assess their safety under REACH, including the use of alternative methods to direct testing, such as grouping and read-across (European Chemicals Agency (ECHA), 2012, European Chemicals Agency (ECHA), 2017b, European Chemicals Agency (ECHA), 2017c).

At the global level, the Organisation for Economic Co-operation and Development's (OECD) Working Party on Manufactured Nanomaterials (WPMN) has reacted to advancements in scientific knowledge concerning NMs, for instance by assessing the applicability of existing OECD Test Guidelines (TGs) for the determination of physicochemical and (eco)toxicological properties of chemicals, and the need to adapt them or develop new TGs for NMs (OECD, 2018; Rasmussen et al., 2016, Rasmussen et al., 2018). The International Organization for Standardization (ISO) and the European Committee for Standardization (CEN) have also contributed to the field through a number of standardisation documents (CEN, 2018; ISO, 2018).

Recent scientific reviews (Liguori et al., 2016; Nowack, 2017; Burello, 2017; Fadeel et al., 2018; Erbis et al., 2016; Hristozov et al., 2016; Oomen et al., 2018; Trump et al., 2018) have described the state-of-the-art and trends in nanoEHS tool development by all actors, including researchers in the academia and industry, regulators and policy-makers worldwide. These reviews tend to focus on the available tools and frameworks and discuss their regulatory maturity, i.e. whether certain tools are adequate and sufficiently reliable for the purposes of regulatory risk assessment. Some reviews emphasise specific areas of NM safety assessment, such as control banding (Liguori et al., 2016), exposure modelling (Nowack, 2017) or toxicity prediction and analysis (Burello, 2017; Fadeel et al., 2018). Others compare traditional regulatory procedures with alternative frameworks and decision schemes, concluding on their strengths and limitations or calling for a different way of governing nanotechnologies (Erbis et al., 2016; Hristozov et al., 2016; Oomen et al., 2018; Trump et al., 2018). A few authors attempt to extensively collect or discuss tools that are available to implement every aspect of the safety assessment prescribed in a regulatory context, i.e. from NM identification and physicochemical characterisation to risk estimation and management, or that may help manufacturers and regulators in prioritisation and early decision-making (e.g. control banding, decision support systems) (Hristozov et al., 2016; Steinhäuser & Sayre, 2017). However, these papers simply list the reviewed tools or categories of tools in summary tables including short descriptions, literature references and some recommendations for use.

This work presents the first-ever inventory of more than 500 publicly available tools that can support NM safety assessment, as prescribed in legislation and in less data-intensive paradigms. The inventory is as complete as possible and aligned with the NANoREG Framework (Gottardo et al., 2017), collecting tools to implement the regulatory provisions under REACH as well as safe-by-design (SbD), risk prioritisation and assessment (nanoRA), and life cycle assessment (LCA) for NMs. These three strategies seek to facilitate and accelerate achieving the REACH objectives (health and environment protection while ensuring free movement of substances, competitiveness and innovation) while demanding less testing effort from the industry than the current safety assessment procedure according to legal requirements does, and promoting a more predictive and integrative approach by regulators (Gottardo et al., 2017).

The NANoREG Toolbox (Jantunen et al., 2017), developed with support from the EU seventh framework programme (FP7) project NANoREG, synthesises information about the available tools in a publicly and freely accessible Microsoft Excel®-based descriptive metadata set. It can be downloaded and exploited by all stakeholders in the nanoEHS field: scientists, industry, non-governmental organisations and regulators. As the Toolbox addresses all nanoEHS aspects, many diverse areas of safety assessment and stakeholder needs can be covered by using it as a starting point to search for available tools. It primarily collects tools that are publicly available and ready for use, and organises them in worksheets mirroring the structure of the NANoREG Framework. It also keeps track of a selection of tools that are known to be close to release, based on communications from the developers. For each tool, up to ten information items (e.g. purpose, regulatory status) have been collected. The Toolbox also provides the link to the webpage or other source where the tool can be downloaded or otherwise accessed. The inventory thus works as a reference metadata set that provides information relevant to helping the users find the right tool(s) for their purpose(s).

This article describes and discusses the structure and content of the Toolbox. The concepts used and the criteria for tool collection are specified in Section 2 (Methods), whereas the ways the tools and related information items have been organised in the Excel® files are explained in Section 3.1 (Toolbox structure). The article also provides a quantitative overview of the tools stored in the Toolbox at the time of publication (Section 3.2). As an example of how the Toolbox can be used and what types of tools can be found in it, Section 3.3 focusses on the tools available to implement the EC Recommendation.

Results are discussed in Section 4. This article does not aim to use the content of the Toolbox to identify and discuss research gaps or further needs for tool development or validation in the nanoEHS field in any detail, as this has been more extensively done e.g. in the NANoREG Framework (Gottardo et al., 2017) and by Sayre et al. (2017). Neither does it assess the scientific robustness of the available tools, beyond noting that either the recorded tools have passed scientific peer review or institutions have associated their name with them. However, certain evident gaps and other observations are noted. This article also reports the fraction of tools that have undergone a formal validation or standardisation process, useful as an indication of the regulatory maturity.

It should be noted that while the NANoREG Toolbox is organised according to the logic of chemical and NM safety assessment in the EU (REACH), this is highly consistent with the international framework of chemical risk assessment (WHO, 2001; OECD, 2012) and with the general understanding of information needs regarding chemicals (Organisation for Economic Co-operation and Development (OECD), 1982, Organisation for Economic Co-operation and Development (OECD), 1987) and NMs (OECD, 2010; Stefaniak et al., 2013). Therefore, the NANoREG Toolbox presents tools that serve the safety assessment of NMs independently from the location or legislative framework where the assessment is required.

## 2 Methods

### 2.1 Concepts

For the purposes of the NANoREG Toolbox (Jantunen et al., 2017), a ‘tool’ is defined as “*an experimental*, *computerised*, *or decision procedure used for generating*, *collecting*, *assessing*, *and*/*or storing a certain type of output*” (modified after Gottardo et al., 2016). A tool either i) directly addresses or supports addressing an endpoint in the process of assessing the safety of NMs (e.g. a specific physicochemical or toxicological property, the prediction of exposure levels in water or soil) or ii) is necessary or helpful in the process of addressing one or several endpoints (these tools include e.g. dispersion protocols and data quality assessment tools). In the context of this article, the latter are referred to as ‘support functions’.

Tools that are currently publicly available and ready for use are named ‘functional tools’. Conversely, ‘Prospective tools’ have not yet been fully developed or published at this time. The latter are expected to become publicly available in the short to medium term, based on public claims by the developers or personal communications from the developers to the authors of this article.

It is worth noting that while the functional tools recorded in the NANoREG Toolbox are ready for use, their features may be more or less developed. For example, a model and its algorithms may have been comprehensively described in a scientific publication, allowing a skilled user to recreate the model and then use it to produce data or information, or the model may be available as a(n online) tool with a graphical user interface, or the user-friendliness of the model may lie somewhere in between.

Although the recorded functional tools are publicly available, using them may require registration as a user or payment. However, services and technical instruments were excluded from the NANoREG Toolbox. The exclusion of services, such as those provided by consultants (who may e.g. use software in their possession on behalf of a client to produce data or information), ensures that the recorded tools are *directly* accessible to the general public and therefore can, in principle, be used and analysed by anyone. Instructions on using technical instruments (for making measurements to fulfil data requirements) have been included only if such instructions concern measurements that specifically address NMs (e.g., Clavaguera et al., 2016; Mast & De Temmerman, 2016).

The NANoREG Toolbox provides a number of information items (listed under Section 3.1 of this article) as descriptors of each tool. In the following, certain descriptors are explained as concepts in the context of this inventory.

The ‘Purpose’ of a tool is the endpoint relevant to NM safety assessment served by the tool, such as measuring particle size number distribution, characterising occupational exposure or grouping and read-across. Each tool is assigned to a ‘Type’ by choosing one of the following six options: ‘experimental protocol’, ‘model’, ‘decision support tool’, ‘guidance’, ‘report’, ‘data management tool’, or ‘repository’ (Jantunen et al., 2017).

The ‘Regulatory status’ of a tool indicates its level of regulatory maturity and acceptance (Jantunen et al., 2017). The main options for the regulatory status range from ‘research product’ to ‘validated’, ‘harmonised’ or ‘standardised’. A tool is a research product when it has not been validated or has an unclear validation status, based on the available information. A tool can be considered validated when both reliability and relevance have been established for the specific purpose of NM safety assessment, according to a formal and scientifically sound procedure (Gottardo et al., 2016). Within the NANoREG Toolbox, an experimental protocol is recorded as validated if it has undergone both intra- and inter-laboratory validation, but has not been standardised or harmonised by a dedicated international body. Standardisation of tools is performed by e.g. ISO or CEN, whereas harmonisation refers to the TGs developed, agreed and published by the OECD for producing data to fulfil regulatory requirements. Harmonised tools are mutually accepted by countries adhering to the Mutual Acceptance of Data (OECD, 1981).

These four main options – research product, validated, harmonised or standardised – do not apply to all of the recorded tools. Therefore documents providing recommendations of regulatory relevance (developed by a competent authority) were assigned the status of ‘regulatory document’, while the ‘not applicable’ option indicates that, in these cases, the regulatory status concept was not considered meaningful.

### 2.2 How tools were collected and described

Both functional and prospective tools were collected from the available literature, by Internet searches (e.g. Scopus, Google) and via input by NANoREG project partners. Importantly, the discovery of a tool often led to finding others by association. Tools developed both within NANoREG and by other initiatives, also beyond Europe, were considered. For each tool, the detailed information needed for this inventory was obtained from the relevant references (publications and websites) or by directly contacting the organisations or persons responsible for the tool. The content of the Toolbox is the outcome of a review process based on the authors' expert judgment that entailed compromises between completeness and accuracy on the one hand, and pragmatism on the other (see Section 4.1).

The collected tools are described on the basis of the information provided by the developers in relevant publications or communications. This information was also used by the authors to assign each record a purpose, type and regulatory status (see Section 2.1), bearing in mind which step or approach in the NM safety assessment process each tool actually supports according to the structural logic of the Toolbox (see Section 3.1). Where available, literature in which the tool has been used in a case study or that discusses a tool's characteristics, such as its scientific robustness, was recorded as references in the corresponding record under the dedicated fields ‘Publication(s)’ or ‘Other information’ (see Section 3.1).

## 3 Results

### 3.1 Toolbox structure

**Fig. 1:**
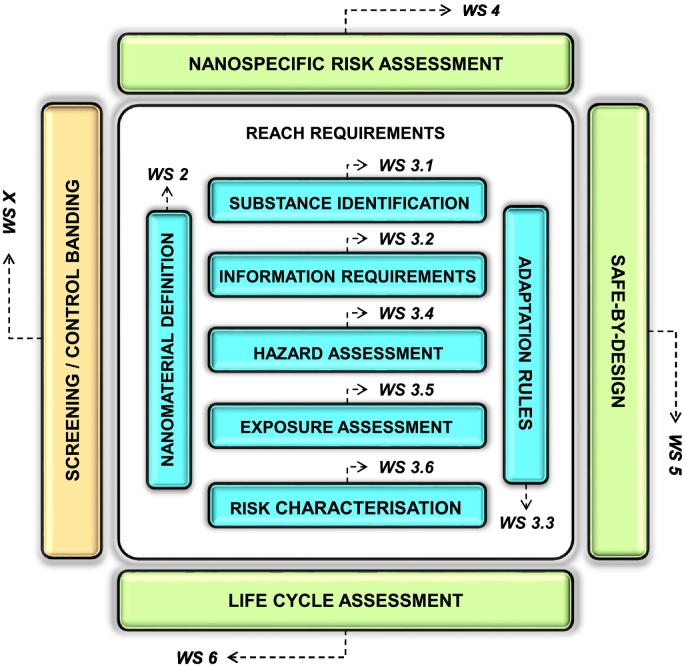
NANoREG Toolbox structure: the two Excel® workbooks are organised into eleven worksheets (WS) depicted as coloured boxes. Each WS is identified by a number (given close to each box) and a title (written in each box) reflecting their content. The numbers refer to the corresponding sections of the NANoREG Framework (Gottardo et al., 2017). These WSs provide an overview of the tools useful for implementing the two Parts (I and II) of the NANoREG Framework. The blue rectangles refer to Part I, which addresses the regulatory requirements on safety assessment (mainly in REACH) and discusses their implementation for NMs. The green rectangles refer to Part II, which proposes three forward-looking strategies that may facilitate or accelerate the implementation of those requirements. The yellow rectangle refers to tools with other purposes related to NM safety assessment.

The NANoREG Toolbox consists of two Excel® workbooks, named ‘Toolbox’ and ‘Prospective tools’, saved as separate .xlsx files. ‘Toolbox’ contains functional tools only. Each workbook comprises eleven data-containing worksheets (indicated as WSs in Fig. 1) dedicated to different steps or components of the NM safety assessment process. Ten of the worksheets correspond to specific sections of the NANoREG Framework (Gottardo et al., 2017), each of these identified by the corresponding section number and a title indicating the section content. These worksheets provide an overview of the tools useful for implementing both parts of the NANoREG Framework: Part I addresses the regulatory requirements (mainly under REACH) and the related ECHA guidance for NM safety assessment, whereas Part II describes three forward-looking strategies (SbD, nanoRA, and LCA) that aim to achieve the REACH safety objectives in alternative ways (see Section 1). Tools implementing Part I are stored in WS 2 and WSs 3.1–3.6 of the workbooks (blue rectangles in Fig. 1), while those implementing Part II can be found in WSs 4–6 (green rectangles in Fig. 1). The eleventh worksheet records tools for screening, categorisation, prioritisation and control-banding approaches. Such tools do not generate data that meet the regulatory requirements for safety assessment, but they enable risk assessment and management at an earlier stage and when limited information is available (WS X: yellow rectangle in Fig. 1). Although the NANoREG Framework did not specifically cover these approaches, these tools were included in the Toolbox due to their relevance and utility in the context of NM safety assessment. Both workbooks also contain an introductory worksheet (WS 1) providing definitions of the main concepts and clear indications on how to use the Toolbox for either consultation or further collection of tools.

The worksheets are listed below:

• ‘WS 1 About the NANoREG Toolbox’ contains general information on the context and structure of the Toolbox and explanations of the concepts used;
• ‘WS 2 EC Nano Definition’ is dedicated to tools for determining if the material under assessment fulfils the EC Recommendation (see Section 1);
• ‘WS 3.1 REACH Substance ID’ is dedicated to tools for determining substance identity parameters for NMs;
• ‘WS 3.2 REACH Info Requirements’ is dedicated to tools for determining physicochemical and (eco)toxicological parameters of NMs according to the current standard information requirements;
• ‘WS 3.3 REACH Adaptation Rules’ is dedicated to tools for applying the rules for adapting the standard testing regime (e.g. grouping, read-across) to NMs when testing is unnecessary or not possible (REACH Annex XI);
• ‘WS 3.4 REACH Hazard assessment’ is dedicated to tools for identifying and assessing the hazards of NMs;
• ‘WS 3.5 REACH Exposure assessment’ is dedicated to tools for defining exposure scenarios and assessing exposure to NMs (including exposure control tools);
• ‘WS 3.6 REACH Risk characterisation’ is dedicated to tools characterising the risks of NMs (including risk management tools);
• ‘WS 4 Nanospecific Risk Assessment’ is dedicated to tools implementing the nanospecific prioritisation and risk assessment approach (nanoRA) developed within NANoREG, and other NM-specific approaches to risk assessment;
• ‘WS 5 Safe-by-Design (SbD)’ is dedicated to tools implementing the SbD concept for NMs;
• ‘WS 6 Life Cycle Assessment (LCA)’ is dedicated to tools applying LCA to NMs and combining it with risk assessment; and
• ‘WS X Screening and control banding (CB)’ is dedicated to tools for screening, ranking, prioritizing and classifying the risks of NMs and applying CB to NM risk management.

**Fig. 2:**
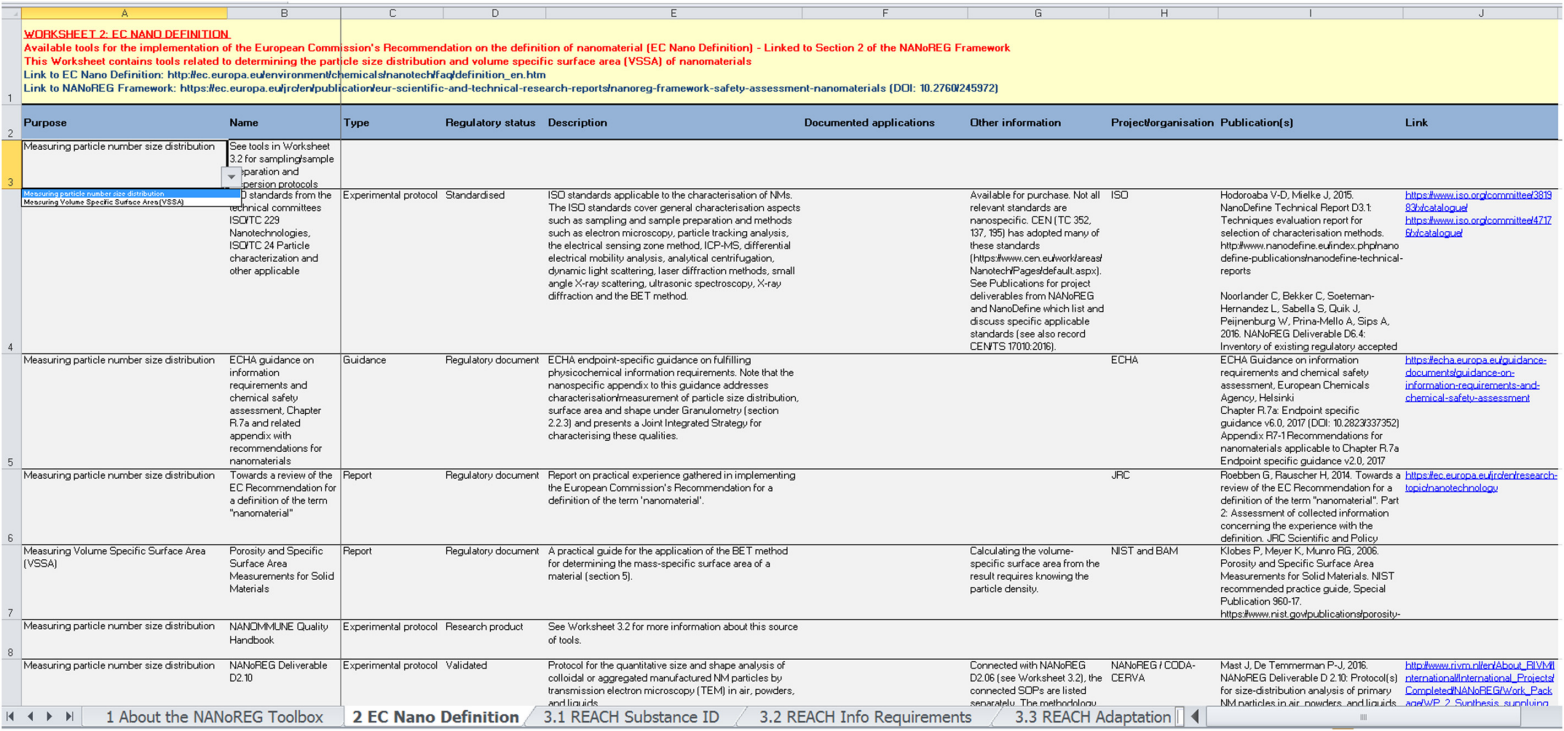
A screenshot from ‘Worksheet 2 EC Nano Definition’ of the Toolbox workbook, with an opened drop-down menu for the purpose of the tool.

A ‘record’ in the Toolbox corresponds to a row in Microsoft Excel® and contains information concerning one or more individual tools. In both workbooks, each WS presents a table where rows correspond to tool records and columns to the various information items concerning the tool(s) covered by each record (see Fig. 2 for an illustrative screenshot of a WS). There are up to ten information items: ‘Purpose’, ‘Type’ and ‘Regulatory status’ (defined in Section 2.1), ‘Name’, ‘Description’, ‘Documented applications’, ‘Other information’, ‘Project/organisation’, ‘Publication(s)’ and ‘Link’. For three information items – Purpose, Type and Regulatory status – drop-down menus with fixed options are used (see Fig. 2). Elsewhere, information is entered as free text.

Purpose, given in the first column, is intended as the main search term for retrieving tools of interest. The drop-down menu for Purpose has been developed individually for each worksheet to reflect the needs for tools within the corresponding component of the NM safety assessment process. The user can apply built-in Excel® data sorting or filtering options to this or any other column to tailor the search of tools within a worksheet for specific needs. Purpose, Type, Regulatory status, Description and either Publication(s) or Link are reported in every record. For the functional tools (in the Toolbox workbook), access information (under Publication(s) or Link) is essential for demonstrating, and keeping track of, the availability of each tool. In the Prospective tools workbook, the expected progress and release schedule of each tool is recorded under Other information, as are any relevant contact details for potential inquiries.

### 3.2 Toolbox content

Many records actually contain information on more than one tool: a record may, for instance, refer to a collection of experimental protocols that can be downloaded from a single website. Hence, the total number of individual tools covered by the information stored in the Toolbox workbook is significantly larger than the number of records. We estimate the number of currently available individual tools to be 544 (for information on how the tools were counted and the detailed counts, see the Supplementary material file).

It should be noted that there are no clear criteria for what constitutes an individual tool. Many tools consist of numerous modules, routines or protocols to be used either separately or in combination with each other. In each case, the view of the tool releasers on what constitutes ‘a tool’ was followed. Some tools occur in several worksheets, as they can support different assessment steps (e.g. a tool that can simultaneously characterise several properties of nanoparticles may support either identifying a material as a NM according to the EC Recommendation (WS 2) or determining substance identity parameters for NMs under REACH requirements (WS 3.1)). To avoid unnecessary repetition, in these cases most of the information about each individual tool is found in one worksheet only. The other occurrences explicitly redirect the user to the record in that worksheet.

Three quarters (75%) of the individual tools covered by the Toolbox workbook are specific to NMs (‘nanospecific’). The rest address either chemicals or emerging technologies in general and are considered applicable to NMs by the tool authors or in relevant guidance.

**Fig. 3:**
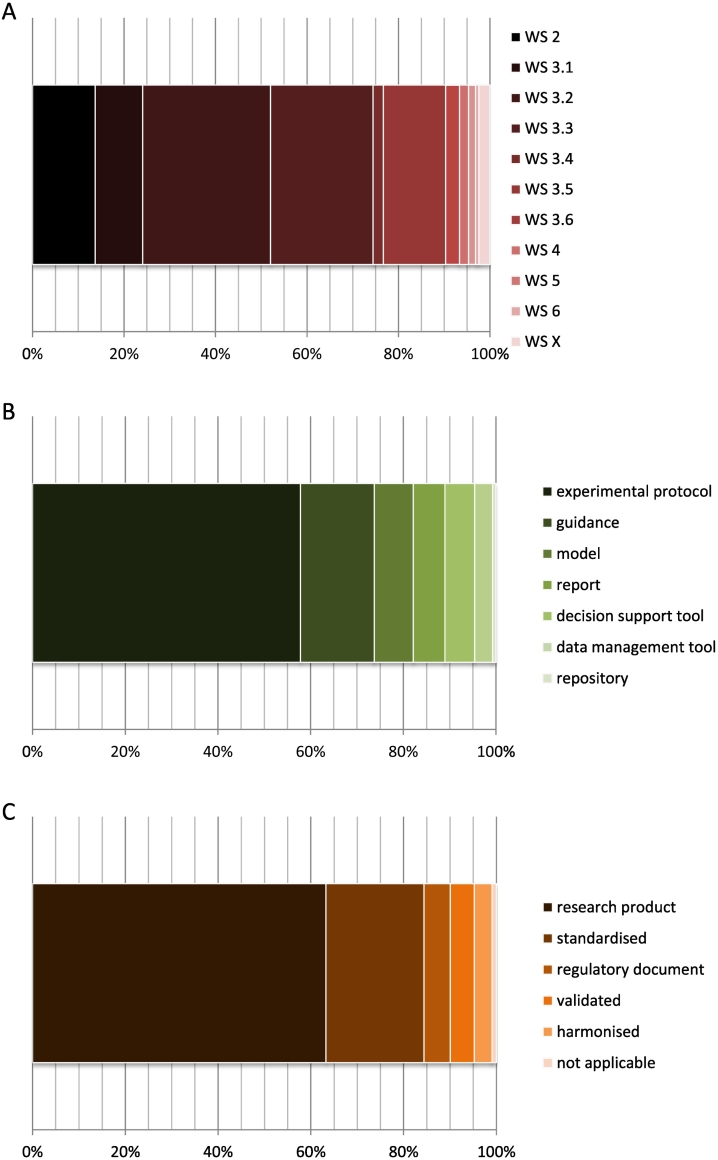
Relative share (%) of individual tools referred to by the Toolbox workbook (N = 544) by worksheet (A), type (B), and regulatory status (C). WS 2: EC nano definition, WS 3.1: REACH Substance ID, WS 3.2: REACH Info Requirements, WS 3.3: REACH Adaptation Rules, WS 3.4: REACH Hazard Assessment, WS 3.5: REACH Exposure Assessment, WS 3.6: REACH Risk Characterisation, WS 4: Nanospecific Risk Assessment, WS 5: Safe-by-Design, WS 6: Life Cycle Assessment, WS X: Screening and C(ontrol) B(anding) (see also Fig. 1).

As illustrated in Fig. 3-A, about 93% of all the individual tools covered by the Toolbox workbook can be used in one of the steps required for the NM safety assessment under REACH (WSs 2 and 3.1–3.6). The most common use of a functional tool is in fulfilling standard information requirements via physicochemical and (eco)toxicological testing (28%), followed by adapting the standard testing regime to produce the required information by alternative methods, such as *in vitro* testing, grouping and read-across, or (Q)SAR models (22%). Determining whether a material fulfils the EC Recommendation (EC, 2011) is addressed by 14% of the tools.

The most common tool type is experimental protocol (58%), followed by guidance document (16%) (Fig. 3-B). However, for many standardised or harmonised test methods and guidelines, it is difficult to draw the line between experimental protocols and guidance documents.

A majority (63%) of the tools recorded in the Toolbox workbook are research products (Fig. 3-C). It should be kept in mind that this is the default regulatory status of most types of tools. Twenty-one percent of the tools qualify as standardised – they have undergone a formal standardisation process, e.g. at ISO or CEN – and 5% are considered validated in the context of this Toolbox, which in the case of experimental procedures indicates that they have undergone a formal inter-laboratory validation procedure with available documentation (Fig. 3-C).

**Fig. 4:**
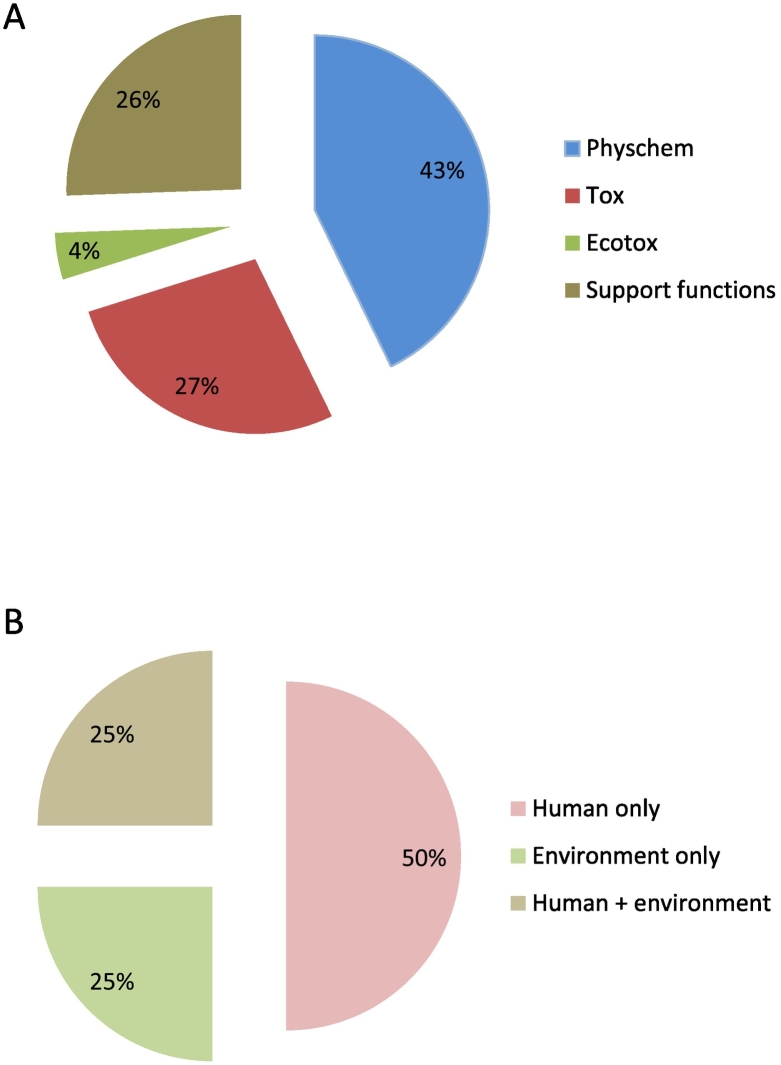
Relative shares (%) of tools addressing certain assessment endpoints covered in specific worksheets of the Toolbox workbook: (A) for WS 3.1 REACH Substance ID, WS 3.2 REACH Info Requirements and WS 3.3 REACH Adaptation Rules (N = 399), by nature of endpoint (physicochemical, toxicological, ecotoxicological or support function), and (B) for WS 3.4 REACH Hazard assessment, WS 3.5 REACH Exposure Assessment and WS 3.6 REACH risk characterisation (N = 104), by protection goal (human health only, environment only, or both human health and the environment).

A large fraction of the categorised individual functional tools address endpoints of physicochemical or toxicological nature (70% of tools in WSs 3.1–3.3; Fig. 4-A) and support human health risk assessment (including hazard, exposure and risk characterisation) (75% of tools in WSs 3.4–3.6; Fig. 4-B), whereas clearly fewer tools address environmental aspects of NMs at the moment. Of all tools addressing human health risk assessment under REACH (WSs 3.4–3.6, N = 82), 56% concern specifically occupational and 9% consumer risk assessment.

As an example of the type of information contained in the NANoREG Toolbox and how it is organised, the contents of worksheet WS 2 on tools supporting the implementation of the EC Recommendation are analysed in detail in the next Section 3.3.

### 3.3 An example: Worksheet 2 – EC Nano Definition

‘WS 2 EC Nano Definition’, in both workbooks, contains information on tools supporting the implementation of the EC Recommendation (EC, 2011) (for an illustrative screenshot, see Fig. 2). The core of this definition states that more than 50% of the primary particles of a NM have one or more external dimensions in the size range 1–100 nm. The particle size number distribution (PSND) is therefore the key parameter to be measured. The definition also mentions the volume specific surface area (VSSA) as a parameter, but its uses are currently limited. While different cut-off values may be applied under other regulatory frameworks (Rauscher et al., 2014; Rauscher, 2017), the PSND – as a metric – and most of the recorded tools, which are not linked to any definition, are relevant also outside of the EU.

As a consequence, the first column (Purpose) in WS 2 has two options in the drop-down menu: i) measuring the PSND and ii) measuring the VSSA. By using the built-in data filter or sorting function in Excel® and choosing, for instance, option i), the user can visualise those WS 2 records that contain information on functional tools for determining the PSND of a material.

The records of the highest regulatory relevance are the harmonised OECD TGs and the documents by ISO and CEN. These may be either guidance documents or experimental protocols; see Supplementary material file for more details. These records refer to selections of individual tools and, as indicated under Other information, the methodology is rarely specific to NMs. For example, the ISO records in WS 2 point to an online catalogue of standards adopted by ISO/TC 229 Nanotechnologies and ISO/TC 24 Particle characterization. Under Other information and Publications, the ISO records refer to two reports developed within the EU-funded projects SIINN ERA-NET (Höhener & Höck, 2015) and NanoDefine (Hodoroaba & Mielke, 2015) and a Technical Specification by CEN (2016). On the basis of literature and experimental evaluation, these three documents together identify no fewer than 39 ISO standards that are considered applicable to determining the PSND of NMs (9 of these standards are nanospecific), and two to measure VSSA (neither of them is nanospecific).

The recorded regulatory documents include guidance texts developed by regulatory authorities, containing recommendations on how to implement certain requirements or perform specific testing on NMs. ECHA has issued endpoint-specific guidance (ECHA, 2016), including a nanospecific appendix (ECHA, 2017b), on how to fulfil the physicochemical information requirements under REACH (including the PSND under ‘granulometry’). The JRC has published two reports (Linsinger et al., 2012; Roebben et al., 2014) containing advice on how to implement the EC Recommendation. Also the US NIST and the German BAM have developed a practical guide (Klobes et al., 2006) concerning the application of the BET method for determining the mass-specific surface area of a material, which, as stated under Other information, can be used to calculate the VSSA.

A number of research products have also been included in WS 2, such as the NanoDefiner e-tool (NanoDefine, 2017), a decision support tool for finding the most reliable measurement method to identify any substance or mixture as a NM or non-NM according to the EC Recommendation (version 1.0.0 of NanoDefiner is publicly available as of November 2017, as recorded under Other information). Other examples include two software instruments relying on image analysis to determine particle size parameters, recorded as data management tools, and a selection of relevant experimental protocols developed by various institutions and projects, including the EU-funded NANoREG, NanoValid and eNanoMapper. The content of the NANoREG deliverable D2.10 (Mast & De Temmerman, 2016) is included in WS 2 both as an individual record – a comprehensive protocol for the quantitative analysis of manufactured NM particles by transmission electron microscopy (TEM) – and as five separate records, each reporting a standard operating procedure (SOP) for the preparation of NM dispersions for TEM investigations or subsequent data analysis (NANoREG D2.10 SOPs 01–05). Under Other information, the record concerning NANoREG deliverable D2.10 cross-references another NANoREG deliverable (D2.06) (Jensen et al., 2016), which is recorded in a different worksheet of the Toolbox workbook (WS 3.2) and concerns the sonication protocols used within NANoREG for NM dispersion in liquid media. As documented by their developers, these NANoREG and the NanoValid SOPs have undergone formal inter-laboratory validation and therefore their regulatory status is set to validated.

More SOPs from other institutions (e.g. the US Nanotechnology Characterization Laboratory) or projects (e.g. the EU-funded NanoDefine, NANOMMUNE and nanOxiMet) are recorded as research products. In the case of nanOxiMet SOPs, the source (DaNa^2.0^, 2018, as recorded under Link) lists the SOPs as validated, but they lack publicly available details or documentation about the validation procedure used and are thus listed in the NANoREG Toolbox as research products. The application domain of the different protocols varies by type of NM and medium, as briefly specified under Description.

Regarding the development of OECD TGs, the 2017 work plan of the TG Programme (OECD, 2017) contains no relevant on-going projects to be included in WS 2 of the Prospective tools workbook (for some examples of such projects, see Prospective tools WS 3.2). However, WS 2 of the Toolbox workbook mentions a proposal by OECD WPMN to develop new guidance on particle size and size distribution determination for NMs (Rasmussen et al., 2018).

Moreover, WS 2 of the Prospective tools workbook records two more decision support tools that the EU-funded project NanoDefine has developed to facilitate the entire process of determining whether a material is a NM according to the EC Recommendation. Public release of these tools is expected in 2018.

## 4 Discussion

### 4.1 Toolbox content

As explained in Section 2.2, the recorded information in the NANoREG Toolbox is based on that provided by the publishers of the tools and any relevant publicly available literature, and could not always be directly verified. However, given the extent and complexity of the Toolbox contents, the authors consider that the inherent uncertainties regarding the reported information on uses, properties, quality and maturity of the recorded tools are acceptable.

A limitation to exhaustively identifying and collecting each and every functional tool available was the fact that releasers of tools relevant for this inventory do not necessarily recognise or publicise them as safety assessment tools. For example, every step of the safety assessment process involves numerous support functions where dedicated tools may have been developed and prove useful, but they have often been published in a different context than NM safety assessment. This makes them difficult to retrieve. Since new tools are also continuously developed and published, completeness was unattainable.

The Toolbox focusses on what tools are available for nanosafety assessment and can also be used indirectly to evaluate the maturity of a research field (e.g. NM physicochemical characterisation) or identify gaps. The number of needed but currently unavailable tools is potentially infinite, particularly since new types of NMs with novel properties are in constant development. It should also be noted that the number of tools available for a specific purpose is not automatically an indication of how well the existing tools cover the current assessment needs. For example, a certain endpoint may be addressed predominantly by modelling either because models are known to produce adequate data for the purpose, or because the absence of suitable methods prevents actual measurements. An existing tool may also be suited for measuring or assessing a certain property of only some well-established type or types of NMs, such as carbon nanotubes or metal-based NMs.

Bearing the above in mind, an analysis of the contents of the Toolbox workbook confirms expected trends in covering the needs in NM safety assessment (Grieger et al., 2012; Selck et al., 2016; Erbis et al., 2016). At this stage, there are indeed more functional tools available for producing physicochemical and toxicological information than for investigating ecotoxicity and environmental fate. Likewise, human health risk assessment is better served than environmental risk assessment, where, due to the challenges of measuring the presence of manufactured NMs in environmental media, fate and exposure assessments are largely based on release assessment and modelling.

This inventory of tools was developed within the NANoREG project, which in itself also contributed to the availability of tools for NM safety assessment, in particular by developing protocols for i) occupational and environmental exposure assessment by measurement (monitoring) and simulation, ii) fulfilling physicochemical and ecotoxicological information requirements and iii) serving various support functions. These contributions can be traced in each worksheet under Project/organisation.

### 4.2 Structure and uses

The NANoREG Toolbox is publicly available and has been released as Microsoft Excel® files, since this format is ubiquitous and affordable. The categorisation of the tools in worksheets that reflect different assessment needs enhances the user-friendliness of the Toolbox. Moreover, the Excel® data filter or sorting function can be utilised to further organise and then select the tools according to their purpose, type and/or regulatory status. Thanks to these features, the NANoREG Toolbox stores a substantial amount of carefully organised information about available tools for NM safety assessment under REACH and beyond, so that stakeholders can easily retrieve the relevant tools that may suit a specific goal.

Every recorded tool is accessible by the general public or, in the case of prospective tools, expected to become so. These tools can therefore globally serve all stakeholders working in the NM safety assessment arena, including legislators, researchers in industry and the academia and non-governmental organisations. The NANoREG Toolbox focusses on fulfilling safety assessment requirements according to REACH. However, the provisions of other EU or non-EU regulatory frameworks concerning NMs, also in other geographical areas, can be similarly supported by the recorded tools. The data and information needs and assessment methodology are reasonably universal (see Section 1), and the tools are rarely tied to a specific regulatory framework. Indeed, many of the recorded tools have been developed by international organisations (e.g. ISO, OECD) or by authorities outside Europe (e.g. the U.S. EPA and NIOSH, Safe Work Australia).

### 4.3 Regulatory status and tool validation

The regulatory status as a descriptor both provides a general overview of the maturity of any specific research field and allows the user to immediately identify the tools that have undergone validation, standardisation or harmonisation, thus having the highest chance of acceptance in a regulatory context (particularly useful for generating data to fulfil the information requirements in a registration dossier). However, the regulatory status offers a snapshot of the current maturity of the tools, which can evolve. A tool (e.g. an experimental protocol) that has not been validated and is now recorded as a research product in the Toolbox may at a later stage be validated and even harmonised or standardised for use in NM safety assessment, to then be mentioned or recommended in guidance provided by regulatory authorities, such as the ECHA, as seen fit.

Particularly for experimental protocols and models, validation is an important step toward adoption as tools for generating data that can be used in a regulatory context. Since the term is used loosely, it should be noted that the NANoREG Toolbox required evidence of a formal validation process meeting certain criteria to categorise an experimental protocol as ‘validated’. The term ‘validation’ also does not necessarily apply to complex environmental fate models, whose performance can merely be tested against observed data in various specific situations (Oreskes et al., 1994); all such models recorded in the Toolbox are currently categorised as research products. In addition to a sufficient scientific robustness of the tool in question, validation requires resources and, in the case of models, data suited for their validation. Formally validated experimental protocols for nanosafety assessment purposes have been recently produced by the EU FP7 projects NanoValid and NANoREG, but they represent a small fraction of the total number of protocols developed by research projects. As recorded by the Prospective tools workbook (e.g. WS 3.2), the OECD plans to develop new TGs and Guidance Documents (GDs) for NMs, for instance for particle size distribution and for dissolution rate in aquatic environments. It can reasonably be expected that more and more protocols will be validated and standardised or harmonised in the near future.

### 4.4 Potential future developments

The NANoREG Toolbox lends itself to conversion into a proper database or digital library and to future expansion. A Wiki-based solution, for instance, would help to keep the metadata set alive by allowing users to add and update records. Also the present format of the Toolbox makes this inventory easy to update (e.g. when a new tool comes to light or the regulatory status of an available tool changes). In developing the database further, the information items purpose, type, regulatory status, description and link (or publication) should be considered compulsory in order to maintain the utility of the records. Thorough user guidance is required to ensure properly formatted and placed records, e.g. the correct assignment of the section (worksheet), tool purpose(s), type and regulatory status.

In the EU, the concept of ‘nano-risk governance’ is being discussed and developed, for instance within the Horizon 2020 project caLIBRAte (caLIBRAte, 2016). Funds have been recently allocated to the further exploration of this topic in the following years, with focus on data and information management, governance tools for NM safety, responsible communication, international cooperation, etc. (EC, 2018b). In this broader context, the NANoREG Toolbox can play a role as a provider of well-organised information about already available tools for NM safety assessment and management within and beyond the regulatory context. The dataset already contains tools for purposes such as data management (WS 3.2), life-cycle assessment (WS 6) and multi-criteria decision analysis (e.g., Subramanian et al., 2016; WSs 3.6 and 4), called for by various authors in the context of governance or safety assessment in the absence of traditional risk assessment tools and data for NMs (e.g., Erbis et al., 2016; Hristozov et al., 2016; Malloy et al., 2016; Fadeel et al., 2018; Linkov et al., 2018; Oomen et al., 2018; Trump et al., 2018). The Toolbox could also easily be extended, for instance by adding new worksheets listing tools for purposes like risk communication or anticipatory activities by regulators (e.g. horizon scanning), which are fundamental components of the governance of emerging technologies (Stone et al., 2017; Linkov et al., 2018).

## 5 Conclusions

The NANoREG Toolbox is an extensive metadata set that makes it easy for any stakeholder to identify and access tools for NM safety assessment. This collection of more than 500 tools is, to the authors' knowledge, an unprecedented effort of inventorying and categorising tools in the nanoEHS field. While it primarily addresses the requirements of the EU's chemical legislation, the recorded tools were in many cases developed outside Europe and apply in a far wider regulatory context. The authors encourage the global nanoEHS community to use, adopt, update and extend the Toolbox, possibly as a wiki-based solution. In this way, this snapshot of the current situation could become an up-to-date and harmonised database, maintained by international community effort and benefiting all stakeholders in the field.

Commonly recognised challenges to assessing the safety of NMs according to the traditional chemical risk assessment paradigm include gaps in methodology and data availability, accentuated by the constantly increasing variety of novel NMs requiring assessment. Efforts for the governance of nanotechnology have therefore called for more integrative ways to assess and achieve safety, including approaches such as safe-by-design, life cycle assessment and multi-criteria decision analysis, and to improve data sharing and risk communication. The NANoREG Toolbox already contains information on existing tools for such activities and can easily be extended to accommodate new types of governance instruments.

## Appendix A Supplementary data

Supplementary material Image 1
