# Supplementary material for: An inventory of ready-to-use and publicly available tools for the safety assessment of nanomaterials
Source: NanoImpact. 2018 Oct;12:18–28. doi: 10.1016/j.impact.2018.08.007 (PMC6255795; doi:10.1016/j.impact.2018.08.007)
Supplement: Supplementary file 1 — Supplementary material [file mmc1.pdf]

Supplementary Material: How the individual tools were counted

To manuscript:

Jantunen, A.P.K. *et al*. An inventory of ready-to-use and publicly available tools for the safety assessment of nanomaterials.

Many of the records of the NANoREG Toolbox workbook represent sources of tools rather than individual tools. For the full statistics regarding the individual tools covered by this workbook, all relevant individual tools were counted according to the purpose served (as recorded in column A of the Toolbox workbook). All tool counts per purpose differing from 1 in the Toolbox workbook are detailed in the Table SM-1 below, and the counts of individual tools per each worksheet and tool type are given in Tables SM-2 (Toolbox) and SM-3 (Prospective tools). The Prospective tools records include no tool sources, and therefore one record always covers only one tool.

From sources providing several individual tools, we counted only those (such as individual SOPs) that address an endpoint relevant to information requirements under REACH or help to decide whether or not a material is a nanomaterial (NM) according to the current definition in the EC Recommendation (EC, 2011). A given tool may address such an endpoint directly or facilitate addressing at least one endpoint (so-called 'support functions'). Since NMs and information requirements specific to them are at present not explicitly addressed in the EU REACH Regulation, the relevance of individual tools was judged on the basis of current ECHA guidance (ECHA, 2008, 2011a–d, 2017a–g) addressing either chemicals in general or NMs specifically.

Regarding ISO, ASTM and EN standards for deciding whether or not a material is a NM according to the EC Recommendation (WS 2), or for fulfilling information requirements under REACH (WSs 3.1–3.3), we counted the individual standards that were named in one or more of three publications (Hodoroaba & Mielke, 2015; Höhener & Höck, 2015; CEN, 2016) that discuss the 'nano' relevance of

available standards or in current ECHA guidance that addresses NMs specifically (ECHA, 2017a,c,e,g).

Regarding OECD Test Guidelines (TGs), we counted TGs either already published by the OECD as developed specifically for NMs or adapted for NMs, or judged as currently applicable to NMs in communications from the OECD secretariat (based on OECD, 2009, 2012, 2014a–c, 2016; Hoffman et al., 2016; Hund-Rinke et al., 2016).

Regarding NANOMMUNE protocols, we counted the REACH-relevant protocols contained by the online Quality Handbook document from this project (Krug, 2011).

Regarding Nanotechnology Characterization Laboratory (NCL, 2017) and National Institute of Standards and Technology (NIST, 2017) protocols, and NanoBRIDGES (NANOBRIDGES, 2017; STC Lab, 2017a,b) and nanoinfo.org (Nanoinfo.org, 2017) models, we counted the REACH-relevant protocols or models covered by their website or associated websites as of July 2017.

It should be noted that where the same individual tool (e.g. a certain standard as an experimental protocol) serves several different purposes, either within the same worksheet or in different worksheets, such overlap was accounted for in the tool counts. In practice, a reoccurring tool was counted only once within a given worksheet and only once for the total count of tools of different types covered by the workbook. For calculating the percentages of tools in different worksheets of the workbook, any tool occurring in more than one worksheet was divided equally among these worksheets. In the Prospective tools workbook, overlap between worksheets does not occur.

Table SM-1. All tool counts differing from 1 in the Toolbox workbook.

Worksheet	Record	Purpose	#
2	ISO standards	PSND	39
		VSSA	2
	ASTM standards	PSND	2
	EN standards	PSND	3
	OECD TGs	PSND	0
	OECD reports	PSND	3
	NANOMMUNE SOPs	PSND	3
	NCL SOPs	PSND	5
3.1	ISO standards	Shape	10
		Aspect ratio	7
		Chemical characterisation	27
	NANOMMUNE SOPs	Chemical characterisation	2
	NCL SOPs	Shape	2
		Chemical characterisation	2
3.2	ISO standards	Water solubility	3
		Crystalline phase	5
		Zeta potential	3
		Physicochemical properties (incl. mass, density, viscosity)	20
	OECD TGs	Reproductive toxicity	4
		Repeated dose toxicity	3
		Mutagenicity	5

	OECD reports	Physicochemical properties	2
	NIST SOPs	Sampling/sample preparation	2
		Dispersion protocol	2
		Experimental exposure characterisation	4
		Ecotoxicity	2
	NANOMMUNE SOPs	Toxicity	8
	NCL SOPs	Zeta potential	3
		Toxicity/ecotoxicity	7
		Experimental exposure characterisation	2
3.3	NanoBRIDGES models	QSARs	21
	NANOMMUNE SOPs	In vitro testing	28
	NCL SOPs	In vitro testing	31
	NANoREG / Institute of Protein Biochemistry SOPs	In vitro testing	3
3.5	Nanoinfo.org	Characterising environmental exposure	2
3.6	Nanoinfo.org	Characterising environmental risk	2

Table SM-2. Number of individual tools covered by the Toolbox workbook, by worksheet and tool type. *The total numbers have been corrected to reflect the number of unique individual tools, since some tools appear in more than one worksheet. Note that the worksheet (column) totals have not been similarly corrected and, consequently, do not add up to the overall total. SbD = Safe-by-Design, LCA = Life Cycle Assessment.

Worksheet	Nano definition	Substance identity	Information requirements	Adaptation rules	Hazard assessment	Exposure assessment	Risk characterisation	Nanospecific risk assessment	SbD	LCA	Screening & control banding	Total*
**Guidance**	25	20	19	4	6	10	3	4	2	1	1	86
**Report**	8	3	8	5	0	6	2	1	4	2	0	37
**Model**	0	0	0	26	1	14	6	0	0	0	0	46
**Decision support tool**	1	1	0	0	3	7	7	6	2	1	14	35
**Data management tool**	3	3	6	5	3	1	0	0	1	0	0	21
**Repository**	0	0	0	4	0	0	0	0	0	0	0	4
**Experimental protocol**	46	36	123	78	0	39	0	0	0	0	0	315
**Total**	83	63	156	122	13	77	18	11	9	4	15	544

Table SM-3. Number of individual tools covered by the Prospective tools workbook, by worksheet and tool type. SbD = Safe-by-Design, LCA = Life Cycle Assessment.

**Worksheet**	**Nano definition**	**Substance identity**	**Information requirements**	**Adaptation rules**	**Hazard assessment**	**Exposure assessment**	**Risk characterisation**	**Nanospecific risk assessment**	**SbD**	**LCA**	**Screening & control banding**	**Total**
**Guidance**	0	0	4	0	0	2	0	0	0	1	0	7
**Report**	0	0	0	0	0	0	1	0	1	0	0	2
**Model**	0	0	0	0	1	4	0	0	0	0	0	5
**Decision support tool**	2	0	0	0	0	1	3	1	1	0	0	8
**Data management tool**	0	0	0	0	3	1	0	0	1	0	0	5
**Experimental protocol**	0	0	2	0	0	0	0	0	0	0	0	2
**Total**	2	0	6	0	4	8	4	1	3	1	0	29

References

Drug Theoretics and Cheminformatics Laboratory (DTC Lab), 2017a. Cheminformatics tools, http://dtclab.webs.com/software-tools (last accessed 20 April 2018)

Drug Theoretics and Cheminformatics Laboratory (DTC Lab), 2017b. QSAR Model Development Using DTC Lab, Software Tools, http://teqip.jdvu.ac.in/QSAR_Tools/ (last accessed 20 April 2018)

European Chemicals Agency (ECHA), 2008. Guidance on information requirements and chemical safety assessment, Chapter R.6: QSARs and grouping of chemicals (ECHA, Helsinki). https://echa.europa.eu/documents/10162/13632/information_requirements_r6_en.pdf (last accessed 20 April 2018)

European Chemicals Agency (ECHA), 2011a. Guidance on information requirements and chemical safety assessment, Chapter R.2: Framework for generation of information on intrinsic properties v2.1. (ECHA-2011-G-11-EN, ECHA, Helsinki). https://echa.europa.eu/documents/10162/13643/information_requirements_r2_en.pdf (last accessed 20 April 2018)

European Chemicals Agency (ECHA), 2011b. Guidance on information requirements and chemical safety assessment, Chapter R.3: Information gathering v1.1. (ECHA-2011-G-12-EN, ECHA, Helsinki). https://echa.europa.eu/documents/10162/13643/information_requirements_r3_en.pdf (last accessed 20 April 2018)

European Chemicals Agency (ECHA), 2011c. Guidance on information requirements and chemical safety assessment, Chapter R.4: Evaluation of available information v1.1. (ECHA-2011-G-13-EN, ECHA, Helsinki). https://echa.europa.eu/documents/10162/13643/information_requirements_r4_en.pdf (last accessed 20 April 2018)

European Chemicals Agency (ECHA), 2011d. Guidance on information requirements and chemical safety assessment, Chapter R.5: Adaptation of information requirements v2.1. (ECHA-2011-G-15-EN, ECHA, Helsinki). https://echa.europa.eu/documents/10162/13643/information_requirements_r5_en.pdf (last accessed 20 April 2018)

European Chemicals Agency (ECHA), 2017a. Guidance on information requirements and chemical safety assessment, Appendix R.6-1 for nanomaterials applicable to the Guidance on QSARs and Grouping of Chemicals v1.0 (ECHA-17-G-17-EN, ECHA, Helsinki). http://doi.org/10.2823/884050

European Chemicals Agency (ECHA), 2017b. Guidance on information requirements and chemical safety assessment, Chapter R.7a: Endpoint specific guidance v6.0 (ECHA-17-G-18-EN, ECHA, Helsinki). http://doi.org/10.2823/337352

European Chemicals Agency (ECHA), 2017c. Guidance on information requirements and chemical safety assessment, Appendix R7-1 Recommendations for nanomaterials applicable to Chapter R.7a Endpoint specific guidance v2.0 (ECHA-17-G-16-EN, ECHA, Helsinki). http://doi.org/10.2823/412925

European Chemicals Agency (ECHA), 2017d. Guidance on information requirements and chemical safety assessment, R.7b: Endpoint specific guidance v4.0 (ECHA-17-G-10-EN, ECHA, Helsinki). http://doi.org/10.2823/84188

European Chemicals Agency (ECHA), 2017e. Guidance on information requirements and chemical safety assessment, Appendix R7-1 Recommendations for nanomaterials applicable to Chapter R.7b Endpoint specific guidance v2.0 (ECHA-17-G-15-EN, ECHA, Helsinki). http://doi.org/10.2823/72973

European Chemicals Agency (ECHA), 2017f. Guidance on information requirements and chemical safety assessment, Chapter R.7c: Endpoint specific guidance v3.0 (ECHA-17-G-11-EN, ECHA, Helsinki). http://doi.org/10.2823/43472

European Chemicals Agency (ECHA), 2017g. Guidance on information requirements and chemical safety assessment, Appendix R7-2 Recommendations for nanomaterials applicable to Chapter R.7c Endpoint specific guidance v2.0 (ECHA-17-G-14-EN, ECHA, Helsinki). http://doi.org/10.2823/647499

European Commission (EC), 2011. Commission Recommendation of 18 October 2011 on the definition of nanomaterial (2011/696/EU). Off. J. Eur. Un. L 275, 38–40. http://eur-lex.europa.eu/legal-content/EN/TXT/?uri=CELEX:32011H0696 (last accessed 19 April 2018)

European Committee for Standardization (CEN), 2016. CEN/TS 17010:2016 Nanotechnologies - Guidance on measurands for characterising nano-objects and materials that contain them (CEN, Brussels). https://standards.cen.eu/dyn/www/f?p=204:110:0::::FSP_PROJECT,FSP_ORG_ID:37570,508478&cs=1B118626C371517092C635737273B5E58 CEN/TS 17010:2016 (last accessed 19 April 2018)

Hodoroaba, V.-D. & Mielke, J., 2015. NanoDefine Technical Report D3.1: Techniques evaluation report for selection of characterisation methods, http://www.nanodefine.eu/index.php/nanodefine-publications/nanodefine-technical-reports (last accessed 19 April 2018)

Hofmann, H. *et al*., 2016. NANoREG Deliverable D 2.09: Revised OECD methods for determination of physicochemical NM properties, https://www.rivm.nl/en/About_RIVM/Mission_and_strategy/International_Affairs/International_Projects/Completed/NANoREG/Work_Package/WP_2_Synthesis_supplying_and_characterization

Höhener, K. & Höck, J., 2015. Consolidated Framework for EHS of Manufactured Nanomaterials, ERA-NET SIINN Final report of Deliverable no. D2.6, http://www.siinn.eu/en/the-project-and-results/2-publications/,173.html (last accessed 19 April 2018)

Hund-Rinke, K. *et al*., 2016. Regulatory ecotoxicity testing of nanomaterials – proposed modifications of OECD test guidelines based on laboratory experience with silver and titanium

dioxide nanoparticles, *Nanotoxicology* 10(10): 1442–1447. http://doi.org/10.1080/17435390.2016.1229517

Krug HF (ed.), 2011. NANOMMUNE Quality Handbook: Standard Procedures for Nanoparticle Testing, http://www.nanopartikel.info/files/methodik/NANOMMUNE-Quality-Handbook-SOPs.pdf (last accessed 20 April 2018)

Nanotechnology Characterization Laboratory (NCL), 2017. Assay Cascade Protocols, https://nanolab.cancer.gov/resources/assay-cascade-protocols (last accessed 20 April 2018)

National Institute of Standards and Technology (NIST), 2017. Nano-Measurement Protocols, https://www.nist.gov/mml/nano-measurement-protocols (last accessed 20 April 2018)

NanoBRIDGES, 2017. Software, http://nanobridges.eu/software/ (last accessed 20 April 2018)

Nanoinfo.org, 2017. http://nanoinfo.org/ (last accessed 20 April 2018)

Organisation for Economic Co-operation and Development (OECD), 2009. Preliminary Review of OECD Test Guidelines for their Applicability to Manufactured Nanomaterials (ENV/JM/MONO(2009)21, OECD, Paris). http://www.oecd.org/officialdocuments/publicdisplaydocumentpdf/?doclanguage=en&cote=env/jm/mmon(2009)21 (last accessed 20 April 2018)

Organisation for Economic Co-operation and Development (OECD), 2012. Inhalation Toxicity Testing: Expert Meeting on Potential Revisions to OECD Test Guidelines and Guidance Document (ENV/JM/MONO(2012)14, OECD, Paris). http://www.oecd.org/officialdocuments/publicdisplaydocumentpdf/?cote=env/jm/mono(2012)14&doclanguage=en (last accessed 20 April 2018)

Organisation for Economic Co-operation and Development (OECD), 2014a. Ecotoxicology and Environmental Fate of Manufactured Nanomaterials: Test Guidelines (ENV/JM/MONO(2014)1, OECD, Paris).

http://www.oecd.org/officialdocuments/publicdisplaydocumentpdf/?cote=ENV/JM/MONO(2014)1&doclanguage=en (last accessed 20 April 2018)

Organisation for Economic Co-operation and Development (OECD), 2014b. Report of the OECD expert meeting on the physical chemical properties of manufactured nanomaterials and test guidelines (ENV/JM/MONO(2014)15, OECD, Paris). http://www.oecd.org/officialdocuments/publicdisplaydocumentpdf/?cote=env/jm/mono(2014)15&doclanguage=en (last accessed 20 April 2018)

Organisation for Economic Co-operation and Development (OECD), 2014c. Genotoxicity of Manufactured Nanomaterials: Report of the OECD expert meeting (ENV/JM/MONO(2014)34, OECD, Paris). http://www.oecd.org/officialdocuments/publicdisplaydocumentpdf/?cote=env/jm/mono(2014)34&doclanguage=en (last accessed 20 April 2018)

Organisation for Economic Co-operation and Development (OECD), 2016. Physical-Chemical Properties of Nanomaterials: Evaluation of Methods Applied in the OECD-WPMN Testing Programme (ENV/JM/MONO(2016)7, OECD, Paris). http://www.oecd.org/officialdocuments/publicdisplaydocumentpdf/?cote=ENV/JM/MONO(2016)7&doclanguage=en (last accessed 20 April 2018)
